# MATCHING DYADIC RESEARCH QUESTIONS WITH DYADIC ANALYTIC MODELS

**DOI:** 10.1093/geroni/igad104.1592

**Published:** 2023-12-21

**Authors:** Joshua Novak

**Affiliations:** Auburn University, Auburn, Alabama, United States

## Abstract

Examining shared and common experiences between dyads affords opportunities to answer interesting research questions using dyadic data. Emerging dyadic methodology has expanded the types of research questions researchers can ask and answer. This session aims to equip researchers with knowledge and resources for grant writing, when planning new data collection projects, or when conducting secondary data analyses. Reviewing both cross-sectional and longitudinal methods, we will walk attendees through several types of research questions and match them with appropriate dyadic analytic strategies. These include the Actor-Partner Interdependence Model (APIM), the Common Fate Model, the Dyadic Growth Curve Model (and ALT Model), the Common Fate Growth Curve Model, the Random Intercept Cross-lagged Model, as well as various extensions. We will address accounting for non-independence in dyadic models, along with how to explore substantive dyadic processes. We will review ways that different approaches conceptualize, assess, and examine within and between dyad comparisons. We will also consider ways to address self and other reports of the same construct, such as health congruence/concordance or discordance/difference, as well as perceptions of accuracy or bias, enhancement, and idealization between dyads. Attendees will learn of resources available to learn more about the methods addressed in this session.

